# Managing risk in ski resorts: Environmental factors affecting actual and estimated speed on signposted groomed slopes in a cohort of adult recreational alpine skiers

**DOI:** 10.1371/journal.pone.0256349

**Published:** 2021-08-19

**Authors:** Luis Carus, Isabel Castillo

**Affiliations:** 1 Faculty of Health and Sports Sciences, University of Zaragoza, Huesca, Spain; 2 Faculty of Business and Public Management, University of Zaragoza, Huesca, Spain; Al Mansour University College-Baghdad, IRAQ

## Abstract

**Background:**

Certain weather conditions are clearly harmful, increasing the risk of injury of winter sports participants substantially. The objective of this study was to investigate actual speeds of skiers on signposted groomed slopes and to measure their skill to accurately estimate them with regard to environmental conditions such as visibility, sky cover, snow quality, wind and temperature.

**Methods:**

The data were obtained from a sample of 421 adult recreational skiers taking ski courses. The Pearson correlation coefficient was used to explore the relationship between actual and estimated speed for all participants. Multiple linear regression analysis was used to measure the effect of environmental conditions on both the skiers’ actual speeds and their errors of estimation. Values of 0.05 or less were considered to indicate statistical significance.

**Results:**

The Pearson correlation coefficient between estimated and actual speed was 0.90 (P < 0.001). Skiers underestimated their actual speed on average by 13.06 km/h or 24.1%. Visibility, quality of snow and wind speed were shown to significantly affect both actual maximum speed and estimated speed. Good visibility, grippy snow and calm wind were associated with both the highest actual maximum speed and the lowest ability to estimate it.

**Conclusion:**

Certain environmental conditions are associated with the actual speed at which skiers travel and with their ability to estimate it. Visibility, quality of snow and wind speed seem to influence both actual speed and the ability to estimate it while sky cover and temperature do not. A reinforced understanding of skiing speed on signposted groomed slopes is useful to gain insight into crashes and the mechanisms of resulting injuries, to evaluate means of protection and to devise successful prevention policies in ski resorts.

## Introduction

Risk management in sports facilities aims at minimizing the vulnerability of sportsmen and sportswomen to the probability that human actions or natural hazards, connected to various factors identified with sports activities, will lead to unwished effects [1]. It involves the identification and assessment of environmental and behavioural risk factors and the implementation and control of injury prevention and safety strategies intended to mitigate the damaging outcomes of accidents in sports [2].

With regard to snow sports, skiing is a mass amateur sport associated with a risk not only to the indemnity of skiers, to a point where its rate of casualties demands permanent on-site emergency services, but also to the ski resorts’ success [3]. The inherently undesirable harm to the skier; frequent lawsuits arising from accidents occurring within resorts’ skiing domains, alleging damages or injuries as a result of negligence or imprudence on their part; the far-from-negligible financial repercussions of insurance premiums; the severe detriment to the resorts’ image, even when they are not found responsible, as the result of negative publicity that reaches a huge audience in the mass media whenever serious accidents occur [4]; or an increase in the dropout rate of skiers as a result of a negative perception of their overall resort experience [5], are all reasons that make risk management in ski resorts a crucial issue for all the stakeholders implicated (skiers, ski resorts, legislators, researchers, etc.).

Past research has shown how skiers’ risk and severity of accidents on signposted groomed slopes depend on their behaviour [6] and, particularly, on their speed immediately preceding them [7]. The fact that kinetic energy of a body in motion increases as the square of its speed, together with the fact that increases in speed adversely impact the responsiveness of the skier to bypass obstacles or other skiers [8,9] can be said to be main reasons why ski casualties are most frequently the result of high-velocity crashes [2].

Consequently, risk management in ski resorts must needs include strategies purposely devised to avoid immoderate speed while skiing [10], especially when, according to Shealy et al. [11], skiers’ mean speed on ski slopes increased by some 11.5 km/h from the 1970s (32.9 km/h) to 2003 (44.5 km/h), a supplementary increase of ~17.5 km/h was measured by Dickson et al. [5] five seasons later (to ~62 km/h in 2011) and, given the steady improvements in such factors as slope design, grooming, protective apparel and boot and ski technology, increases in speed seem reasonably likely that will continue to occur [11].

Such typical efforts as posting speed limits and ski patrol surveillance, whose presence alone, according to Harley et al. [12, p. 5], “make it unlikely that would produce meaningful reductions in collision likelihood or severity of impacts”, have been found to be generally ineffectual because, among other reasons such as signage being intentionally ignored [13] or a lack of knowledge of existing rules on speed control [14], skiers are just unable to gauge how fast they actually ski without an external prompt [5]. Consequently, even though pioneering research by Pinelly et al. [15] on speed reduction on ski slopes, by combining a priming procedure (technique used to subtly guide behaviour by means of activating mental representations) with conventional prevention devices currently used at ski resorts, yielded moderate but promising results, the prevention of speed-related injuries in recreational alpine skiing should include the avoidance of excessive speed through further strategies.

In this sense, in order to manage velocity efficiently a thorough comprehension of the factors that influence the skiers’ speed and their competence to accurately estimate it, both by themselves and by those searching for effective strategies to influence the skiers’ behaviour, is needed [5,16]. Besides, such comprehension would be of great help to evaluate protective equipment (e.g. helmets, back protectors, or safety nets), whose effectiveness depends on the speed at the time of impact [17,18].

For that purpose, past research has measured, among both adults and children, how actual speed of signposted groomed slopes skiers and their competence to perceive and estimate it is affected by diverse individual demographic and behavioural factors [5,11,16–19]. However, particular weather conditions can alter the skiers’ sense of safety and so modify the likelihood of endorsing safe behaviours [15]. Furthermore, environmental conditions, such as the quality of the snow or visibility, have been shown to influence the occurrence of mountain casualties and the severity of resulting injuries [20–24].

Certain weather conditions are clearly harmful, increasing the risk of injury substantially [25]. Visibility, wind or snow conditions appear to be key factors affecting the risk of injury [21,22]. For instance, the average reaction time of skiers, from the time a sign comes into view to the time needed to respond to avoid an obstacle, is 856 ms in clear visibility; therefore, during adverse weather conditions, there is a need to allow for greater times to react to signage before obstacles [8].

In short, gaining as much insight as possible into the factors that can affect the speed of skiers and their ability to estimate it, i.e. being able to comprehensively evaluate the actual skiing speed according to the skiers’ perceptions, seems of capital importance (e.g. for ski rentals and shops to set binding release values; for interpreting the reported speed of skiers in accident surveys; for researchers to conduct epidemiological studies; or for ski resorts to devise their prevention and emergency policies and to optimally allocate resources to them. Hitherto, previous research has focused on demographic, behavioural or topographic factors, and it is our aim know to add the effect of environmental factors to the existing body of knowledge.

Even though isolated environmental factors such as visibility or the difficulty of slopes, have complemented studies on the impact of individual’s personal factors on actual speed on the slopes [11,17,26], to the best of our knowledge, no previous research has considered a comprehensive array of environmental factors when evaluating skiers’ actual speed and their ability to estimate it.

Therefore, a wider comprehension of the factors affecting recreational skiers’ actual speed and their ability to estimate it demands further research on the role of environmental factors. Accordingly, so as to fill this gap, the aim of this study was to measure how environmental conditions affect recreational skiers´ actual maximum speed and competence to accurately estimate it with regard to visibility, sky cover, snow quality, wind and temperature.

## Materials and methods

Our research was carried out following the approval of the University of Zaragoza research board, and the study was conducted following the rules of the Declaration of Helsinki (revised in 2013). The data were gathered between December 2019 and mid-March 2020 at a major ski resort in the Spanish Pyrenees, from a sample of skiers taking ski lessons. All participants gave their informed consent in order to take part in the experiment.

Inclusion criteria included adult (≥18), according to Ruedl et al. [16, p. 119] classification “more-skilled (advanced and experts)”, men and women alpine skiers. Therefore, minors, beginners and lower-level skiers, and participants in other winter sport modalities, such as snowboarders or telemarkers, were excluded.

The reason why only more-skilled alpine skiers were included in the experiment was that they were fully able to confidently ski from green (easiest [≤10° of inclination]) to red (difficult [≤22° of inclination]) groomed slopes, where the fastest speeds and most skiing injuries (and the severest as a result of high-speed collisions) occur [5,16,17,27]. Their skill level was confirmed on the test run trainees have to make before being finally assigned their true level. All participants wore helmets in accordance with the ski school’s rules.

Stratified random sampling was used for choosing participants to quantitatively and qualitatively represent the population of adult more-skilled skiers (~7,000) that took weekly courses during the season which, on their turn, presented quite similar a gender composition (60% male, 40% female) as that of the ~800.000 people that made up the whole population of skiers that visited the resort during the season.

Level III ski instructors, equipped with smartphones preloaded with a recent version (2019) of a GPS-based snow-sports application for Android (Ski Tracks 1.3.17; Core Coders Ltd.). We had its precision previously validated by comparing Ski Tracks’ results with photocells measurements on a closed racing stadium; the results proved negligible mean time differences with no systematic bias.

Instructors collected the participants’ maximum speed records in their usual day’s instructional activity [28,29]. They had been familiarized with the application, warned about participants trying and skiing faster than usual just because of being recorded and, accordingly, asked to inform participants that the focus of the research was on their usual skiing, though they knew their speed was being recorded [5,28].

Speed records were obtained on a variety of groomed red runs, that fed into blue and green ones (low-difficulty) that provided access to the lifts or easy access to the bottom of the hill (i.e. slow zones or areas of potential congestion), as they moved across the resort. Participants took the smartphones with them while completing a red run at their will and ease and immediately after they had stopped they were asked what they thought their top speed had been and the actual maximum speed recorded by their devices checked. Errors of estimation (EE) were the differences between both figures.

The five environmental factors considered were those covered by the ski resort own weather report and were measured according to the resort’s scales: visibility (good [+1000m]/moderate [500-1000m]/poor [-500m]), sky cover (sunny, overcast or snowy), snow quality (grippy, icy spots or wet), wind (calm [≤10km/h]/moderate [11–35 km/h]/strong [≥36 km/h]) and temperature (not cold [≥0°C]/slightly cold [-1° to -10°C]/cold [≤-10°C]). Data on environmental factors were checked on-site by the instructors, at the precise times when measurements took place, against those contained in the ski resort daily reports made available online on a three-hour basis through its own smartphone application, who registered whatever discrepancy. In order to check parameters not so obvious as sky cover or quality of snow, namely wind speed and temperature, instructors profited from actual instant information available, at the top stations of lifts, which, for safety reasons (freezing points or top wind speed at which lifts can be safely operated) all count on their own thermometers and anemometers located along their respective lift lines.

Since exact figures were not provided by the weather reports, dummy coding was used for the ordinal variables, which produced parameters that, given that our model includes only main effects, were deemed reliable enough. Sets of data, on recorded maximum speeds and estimated speeds and on environmental conditions, were entered into Minitab 19 for Windows (State College, PA, USA) for analysis. The Pearson correlation coefficient was used to examine the relationship between recorded maximum speeds and estimated speeds [11,16,17], and multiple linear regression analysis to estimate the effect of environmental conditions on both the skiers’ recorded maximum speed and their error of estimation. All p-values were two-tailed, and values of 0.05 or less were considered to indicate statistical significance. The variance inflation factors (VIF) were calculated to measure how much the variance of the estimated regression coefficients were increased because of collinearity and to quantify its severity. VIF values below the commonly used cut-off of 5 were considered to discard severe collinearity [30].

## Results

**Fig 1:**
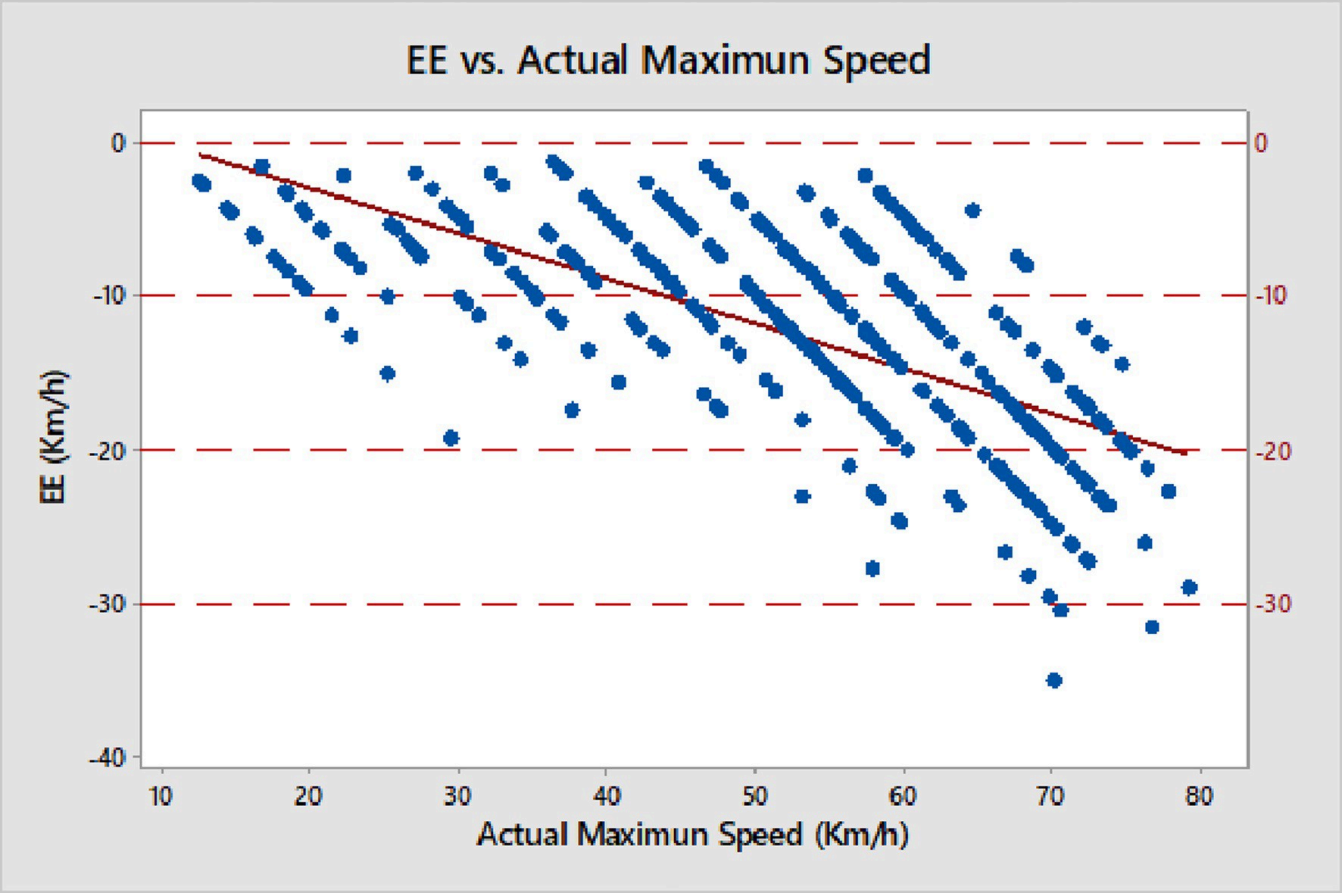
Error of estimation (EE; Y axis) with regard to skiing actual maximum speed (X axis). Parallel lines of data are due to participants estimating their maximum speed in steps of ±5 km/h.

A total of 421 adult “more-skilled” skiers (59% male, 41% female) with a mean (±SD) age of 42.5 (±13.40) years (25.7% from 18 to 30, 15.2% from 31 to 40, 30.9% from 41 to 50, 28.2% older than 50) participated in this study. Mean actual maximum speed and mean estimated speed (±SD) of all participants in all environmental conditions were 54.07 (±15.27) km/h and 41.01 (±11.91) km/h, respectively. The Pearson correlation coefficient between the actual maximum speeds and the estimated speeds was 0.90 (P < 0.001) for all participants. They underestimated their actual maximum speeds, on average by 13.06 (± 6.74) km/h or 24% (Fig 1), which, accordingly, was also their median absolute error of estimation (MAE).

The maximum recorded speed was 79.1 km/h, on a sunny day with good visibility, grippy snow, calm wind and slightly cold temperature, while the minimum was 12.6 km/h, on a snowy day with poor visibility, wet snow, strong wind and cold temperature. The highest error was an underestimation of 66% of the actual speed, while the lowest was an underestimation of 4%. Mean actual maximum and estimated skiing speeds and mean EEs with regard to environmental conditions are presented in Table 1.

**Table 1:** Environmental factors profile and mean values (±SD) of actual maximum speeds, estimated speeds and EEs with regard to visibility, sky cover, snow quality, wind speed and temperature.

Factors	n (%)	Actual maximum speed (km/h)	Estimated speed (km/h)	EE (km/h)*
Visibility				
Good	202 (48)	64.85 ± 7.25	46.71 ± 8.11	-18.14 ± 5.07
Moderate	152 (36)	51.88 ± 7.57	42.95 ± 8.31	-8.93 ± 4.50
Poor	67 (16)	28.64 ± 10.35	21.37 ± 6.01	-7.27 ± 3.39
Sky cover				
Sunny	236 (56)	62.55 ± 10.46	45.68 ± 10.36	-16.87 ± 5.84
Overcast	126 (30)	48.31 ± 10.08	39.76 ± 8.42	-8.55 ± 4.39
Snowy	59 (14)	32.45 ± 13.54	25.00 ± 7.01	-7.45 ± 3.73
Snow quality				
Grippy	261 (62)	58.08 ± 14.63	43.62 ± 11.82	-14.46 ± 6.85
Icy	72 (17)	46.11 ± 16.13	35.13 ± 10.12	-10.98 ± 6.11
Wet	88 (21)	48.69 ± 11.79	37.67 ± 9.23	-11.02 ± 5.69
Wind speed				
Calm	324 (77)	57.06 ± 14.21	43.14 ± 10.99	-13.92 ± 6.87
Moderate	72 (17)	46.93 ± 12.88	35.90 ± 8.84	-11.03 ± 5.36
Strong	25 (6)	35.97 ± 15.50	28.20 ± 11.76	-7.77 ± 4.57
Temperature				
Not cold	46 (11)	48.71 ± 11.82	38.48 ± 9.18	-10.23 ± 5.50
Slightly cold	312 (74)	57.00 ± 14.11	42.95 ± 11.43	-14.05 ± 6.75
Cold	63 (15)	43.50 ± 17.17	33.25 ± 13.27	-10.25 ± 6.13
Total	421 (100)	54.07 ± 15.27	41.01 ± 11.91	-13.06 ± 6.74

* EE, error of estimation. Negative values show an underestimation of actual maximum speeds.

Regarding actual speed, the multiple linear regression analysis, with entering all environmental factors, showed a significant impact of visibility, quality of snow, though it is worth noting that there was very little difference between the influence of icy and wet snow conditions, and wind on actual skiing speed, but not of sky cover or temperature. The values taken by the variance inflation factors of the predictors show that, in principle, they were not correlated (Table 2).

**Table 2:** Results of the multiple linear regression analysis of environmental factors affectation of measured speed.

Factor	B^a^	SE B^b^	t	P value	VIF^c^
Constant	68.17	0.41	65.06	< .001	
Good* vs moderate visibility	-10.32	0.59	-17.64	< .001	1.19
Good* vs poor visibility	-35.96	0.76	-47.36	< .001	1.16
Sunny* vs overcast sky cover	-1.44	1.07	-1.34	.18	3.59
Sunny* vs snowy sky cover	-0.65	1.33	-0.49	.62	3.19
Grippy* vs icy snow	-7.34	0.7	-10.26	< .001	1.09
Grippy* vs wet snow	-6.91	0.67	-10.34	< .001	1.11
Calm* vs moderate wind	-7.15	0.69	-10.30	< .001	1.03
Calm* vs strong wind	-12.19	1.12	-10.92	< .001	1.05
Not cold* vs slightly cold	-1.05	1.11	-0.95	.34	3.51
Not cold* vs cold	-0.94	1.39	-0.68	.45	3.66

^a^ Unstandardized coefficient.

^b^ Standard error of B.

^c^ Variance inflation factor.

* Reference category.

However, even though all VIFs were below the cut-off of 5, it was suspected that sky cover and temperature did not emerge as significant and took noticeably higher VIF values than the rest of environmental conditions because they might be substantially correlated with visibility and wind, respectively. Spearman’s ρ for both pairings–ρ1 = 0.82 (P = 0.000); ρ2 = 0.68 (P = 0.000), respectively–, showed our suspicions justified.

While previous studies have shown certain environmental factors, such as visibility or the quality of snow, to have an impact on actual speed on the slopes [17,23,26], we could find neither previous research on whether sky cover or temperature are associated with the skiers or snowboarders’ actual speed, nor indisputable reasons to support it and, as a result, the opinion of experts (instructors, slope and safety managers, and a meteorologist) was sought.

There was general agreement upon choosing visibility rather than sky cover because it was argued that skiers regulate their speed according to what they can actually see rather than to the sky cover because the latter does not straightforwardly imply visibility, and examples of facts that supported it were provided: when the sky is overcast depending on, for example, the height or density of clouds, the visibility can be good, moderate, poor or even misleading (which is the case in flat light, when slopes become featureless); or when it is snowy the visibility also varies, for instance, depending on the density of the snowfall or even on the size or quality of snowflakes. There was also agreement upon choosing wind rather than temperature because, in a similar vein, they thought skiers speed to be affected, among other reasons such as their control over their skis, by the cold they actually feel, which at a constant temperature varies with the wind speed according to the wind chill index. Accordingly, visibility and wind were chosen as predictors for the models.

The final model of the multiple linear regression analysis explains up to 88% of the variance of the actual speed. In order to account for high R and R^2^ values cross-validation was performed but differences were only obtained in the third and fourth units after de decimal point. Actually, the values of the coefficient of determination barely changed, what led us to believe that the model is dependable enough to make predictions (Table 3).

**Table 3:** Model summary: Effect of visibility, snow quality and wind on the measured speed*.

Model	R	R^2^	Adjusted R^2^	R^2^ change	R^2^ pred	Significant chance	Durbin-Watson
I^a^	0.88	0.77	0.7603	0.76	0.7517	.000	
II^b^	0.92	0.84	0.8452	0.07	0.8440	.000	
III^c^	0.94	0.88	0.8816	0.05	0.8806	.000	1.26

* Dependent variable: Measured speed in km/h.

^a^ Predictors: Visibility.

^b^ Predictors: Visibility, snow quality.

^c^ Predictors: Visibility, snow quality, wind.

In general terms, the results of the multiple linear regression analysis (Table 2) reveal that, as previous research by Shealy et al. [11] had pointed to, in comparison to the actual maximum speed of the groups that skied in better environmental conditions, the groups that were measured in worse environmental conditions skied at significantly lower speeds. Indeed, in comparison to the group that was measured in good visibility, the groups that were measured in moderate or poor visibility skied at significantly lower mean speeds of 10 km/h and 36 km/h, respectively; the group that was measured in calm wind skied significantly faster, 7 km/h and 12 km/h, than the groups that were measured in moderate or strong wind, respectively; and, in comparison to the group that was measured while skiing on grippy snow, those that were measured skiing on icy or wet snow did it at a significantly lower mean speed of ~7 km/h.

With regard to the EE, the results of the multiple linear regression analysis, with entering all environmental factors, are shown in Table 4. They also show a significant impact of visibility, quality of snow and wind on the participant’s ability to estimate their actual maximum speed, but not of sky cover or temperature.

**Table 4:** Results of the multiple linear regression analysis of environmental factors affecting the EE.

Factor	B^a^	SE B^b^	t	P value	VIF^c^
Constant	-18.89	0.35	-53.85	< .001	
Good* vs moderate visibility	8.68	0.49	17.45	< .001	1.13
Good* vs poor visibility	10.26	0.64	15.89	< .001	1.11
Sunny* vs overcast sky cover	0.327	0.910	0.36	.72	3.47
Sunny* vs snowy sky cover	-0.26	1.13	-0.23	.82	3.23
Grippy* vs icy snow	1.77	0.61	2.92	.004	1.01
Grippy* vs wet snow	1.5	0.57	2.64	.009	1.12
Calm* vs moderate wind	1.57	0.59	2.66	.008	1.04
Calm* vs strong wind	3.13	0.94	3.30	.001	1.06
Not cold* vs slightly cold	-0.584	0.942	-0.62	.54	3.49
Not cold* vs cold	-0.86	1.18	-0.73	.46	3.69

^a^ Unstandardized coefficient.

^b^ Standard error of B.

^c^ Variance inflation factor.

* Reference category.

The predictors for the second model were also chosen based on statistical significance and, therefore, sky cover and temperature were not considered. The final model of the multiple linear regression analysis explains 56% of the variance of the EE (Table 5).

**Table 5:** Model summary: Effect of visibility, snow quality and wind on the EE*.

Model	R	R^2^	Adjusted R^2^	R^2^ change	Significant chance	Durbin-Watson
I^a^	0.73	0.53	0.52	0.53	.000	
II^b^	0.74	0.55	0.54	0.02	.000	
III^c^	0.75	0.56	0.56	0.01	.002	2.20

* Dependent variable: Error of estimation (EE) in km/h.

^a^ Predictors: Visibility.

^b^ Predictors: Visibility, snow quality.

^c^ Predictors: Visibility, snow quality, wind.

The results of the multiple linear regression analysis (Table 4) reveal that, in comparison to the groups that made their estimations under better environmental conditions, the groups that made them under worse environmental conditions showed a significantly better ability to estimate their actual speed. Indeed, in comparison to the group that made their estimations in good visibility, the groups that made them in moderate and poor visibility showed a significantly better ability to estimate their actual speed by 8.7 and 10.3 km/h, respectively; the group that made their estimations in calm wind showed a significantly worse ability to estimate their actual speed, by 1.6 km/h an 3.1 km/h, than the groups that estimated their speeds in moderate and strong wind, respectively; and, in comparison to the group that made their estimations while skiing on grippy snow, those that made them while skiing on icy and wet snow showed a significantly better ability to estimate their actual speed by ~1.6 km/h.

## Discussion

The object of this study is to enhance the knowledge of skiing speed through investigating how environmental factors affect adult more-skilled skiers’ speed and their ability to estimate it, with a view to assisting skiers to reassess their behaviours and risk managers to devise and implement preventive and corrective action plans regarding excessive speed on the slopes.

Its main results are that visibility, quality of snow and wind speed seem to impact both actual speed and the ability to estimate it in more-skilled, adult recreational alpine skiers. In comparison to our results, previous research by Dickson et al. [5] reported an average actual maximum speed for adult skiers of 62.06 km/h, which is similar to that obtained in this study for the group of skiers that were measured in sunny days. However, it is noticeably higher than the mean actual maximum speed of all participants in all environmental conditions obtained in this study by ~8 km/h. This visible difference can be due to conspicuously different environmental conditions when measures for both studies were taken, and/or to the previously observed effect that average skiing speed increases as skiing ability increases [11,16–19]. Besides, the aforementioned authors included in their study a higher proportion of professional and highly expert skiers, such as instructors, patrollers or ski racers participating in training days, than there would be in the general ski population.

The opposite effect can be observed when comparing our results to those previously obtained by Ruedl et al. [16] and Bailly et al. [17], who reported an average of maximum speed recorded for skiers of ~45 km/h, which is noticeably lower than that obtained in this study by ~9 km/h. This difference can be also accounted for by different environmental conditions when measures were taken, by the different devices used for measuring purposes [17,31], and/or by their inclusion of “less-skilled” skiers.

With respect to visibility, the difference of 36.21 km/h between the average measured speeds in the worst (“poor”) and the best (“good”) conditions was the greatest difference noted for any of the environmental factors, and translates, for a given mass, into kinetic energy increasing by up to five times. Maximum differences of average measured speeds with regard to the wind speed (21.09 km/h) and quality of snow (9.39 km/h) translate into kinetic energy increasing by 2.5 and 1.6 times, respectively. These results on the behaviour of the skiers regarding speed seem to support that “good weather conditions give skiers a false sense of safety and decrease the likelihood of endorsing safe behaviours” [15, p. 5], what risk managers should do well to keep in mind when devising their prevention and emergency strategies.

Respecting speed perception, our main findings were that skiers in general seem to have a very poor notion of how fast they ski and that environmental conditions, mainly visibility, followed by the quality of snow and wind, appear to have an impact on their ability to estimate speed, in the sense that when environmental conditions worsen skiers show a significantly better ability to estimate it. Our finding that actual speeds were underestimated on average by 13.06 km/h or 24% also differs from the mean error of estimation (11.63 km/h or 19%) observed by Dickson et al. [5]. However, this difference might well be explained again by different environmental conditions when estimations for both studies were made and/or the qualitative composition of the respective samples of participants.

In contrast to previous research by Ruedl et al. [16] and Bailly et al. [17], in our study skiers always underestimated their maximum speed. Besides, the aforementioned authors encountered skiers totally unable to estimate their actual maximum speed by percentages lower than 240% and 300%, respectively, whereas in this study skiers never made errors of estimation exceeding 66% of their actual maximum speed.

Regarding means of protection, no less than 94% of the actual speeds recorded in all environmental conditions were above the 22.3 km/h standard speed set by the American Society for Testing and Materials (ASTM International) for ski helmets [32]. Accordingly, the kinetic energy of the participants skiing between 28.64 km/h (the lowest mean speed recorded in poor visibility) and 64.85 km/h (the highest mean speed recorded in good visibility) was between 1.3 and 2.9 times greater than at the standard specification. Even though, as Carus and Castillo [28] recap regarding their effectiveness, depending on the values taken by a variety of factors that may come together in a collision [33,34], helmets can do their job at speeds above the standard, our findings seem to lay emphasis in the need for the testing protocols and relevant standards to better meet the needs of current skiing speeds.

Also, as regards protective gear, another far from negligible risk deriving from an inaccurate one’s own speed perception is, according to Brunner et al. [18], an incorrect binding setting which, as a result of being either too high or too low, can result in severe injuries. The aforementioned authors found that the varying perception of the individual skiing speed significantly depends on gender, skill level and risk-taking behaviour. Our results now suggest that such environmental conditions as visibility, quality of snow and wind should be added to the list of factors affecting the ability of skiers to estimate their actual speed and, consequently, their accuracy at resetting their bindings when the environmental conditions so require.

With respect to prevention policies, education is one of the ways behaviours may be changed, through helping people become aware of their behaviour, its possible consequences and how to modify it [5]. In this sense, given that, according to Hildebrandt et al. [14], the behavioural patterns of skiers very much depend on their knowledge of current safety rules [35], it follows that in order to lower the number and severity of accidents it is necessary to raise awareness among skiers of the importance of adjusting their speed both to their skill level and to the existing environmental conditions (terrain, weather, traffic, etc.).

Furthermore, all parties involved in a comprehensive ski education (resorts, ski schools, clubs, parents, etc.) should take the necessary steps to make skiers aware of how speed increases the chances of suffering severer accidents and of the advantages of speed self-awareness, and to train them in speed estimation, which, in the words of Ruedl et al. [16, p.122] “could easily be integrated into ski education courses”. Besides, new GPS-based devices, such as smartwatches, navigators or googles, can help skiers to improve their ability to estimate speed and, in general, their understanding of their own behaviour [28,36].

All in all, our findings suggest that, apart from other specific measures adequate for particular environmental conditions (i.e. gear choice and maintenance or use of suitable apparel), whether as a result of good weather altering the skiers’ sense of safety [15] or to bad conditions increasing their risk of injury [22], strategies intended to control speed must be present in all environmental conditions. Information on skiers’ speeds and on how environmental conditions affect their ability to estimate them is instrumental in risk management strategy formulation and selection. Risk managers could well combine identified actual and estimated speeds with prevailing environmental conditions, different groups of skiers and usage patterns of the slopes to implement injury prevention policies for problematic zones within their resorts, such as intersections, elevation changes or areas of heavy traffic.

The main limitation of this study was that in spite of the skiers that made up the sample had been warned that the aim of the study was to investigate their habitual patterns of skiing, they might have tried to ski faster than usual because they knew that their velocity was being recorded. Besides, our sample involved skiers who had made the personal decision to improve their skiing skills, which is an attitude not present in the whole population of ski resorts’ visitors. Finally, our results are principally generalizable to skiers visiting the Spanish Pyrenees, who are for the most part Spanish and French, and may not apply to other nationalities or mountain ranges. To address the study limitations, future research should extend the scope of the investigation in terms of participants characteristics. It may be of interest to research the situation of all skill levels of all snow sports participants, such as snowboarders and freestylers, and those who usually visit ski resorts located in mountain ranges other than the Pyrenees.

## Conclusion

In conclusion, certain environmental conditions are associated with the actual speed at which skiers travel and with their ability to estimate it. Among the factors considered in this study, visibility, quality of snow and wind speed seem to influence both actual speed and the ability to estimate it, in the sense that good visibility, grippy snow and calm wind were associated with both the highest actual maximum speed and the lowest ability to estimate it. A reinforced understanding of skiing speed on signposted groomed slopes is useful to gain insight into crashes and the mechanisms of resulting injuries, to evaluate means of protection and to devise successful prevention policies in ski resorts.

## Supporting information

S1 Dataset Records. (XLSX)
